# Special Issue “Plasticity of the Nervous System After Injury: 2nd Edition”

**DOI:** 10.3390/ijms27177547

**Published:** 2026-08-23

**Authors:** Xavier Navarro, Tessa Gordon

**Affiliations:** 1 Institute of Neurosciences, Department of Cell Biology, Physiology and Immunology, Universitat Autònoma de Barcelona, 08193 Bellaterra, Spain; 2 Centro de Investigación Biomédica en Red sobre Enfermedades Neurodegenerativas (CIBERNED), 28029 Madrid, Spain; 3 Department of Surgery, Division of Plastic Reconstructive Surgery, The Hospital for Sick Children, Toronto, ON M5G 1X8, Canada; tessat.gordon@gmail.com

## 1. Introduction

Regenerative capacity and functional recovery following neural trauma differ between the peripheral nervous system (PNS) and the central nervous system (CNS)**,** driven by striking differences in environmental permissiveness and cell-intrinsic molecular reprogramming. Functional recovery is often poor after peripheral nerve injuries (PNI) despite the capacity of supporting glial cells, the Schwann cells (SCs) that myelinate the axons, to support the regeneration of the injured axons and the reinnervation of target muscle and sense organs. Studies on PNI have revealed multiple cellular and molecular mechanisms involved in the initial Wallerian degeneration and the subsequent axonal regeneration process [1]. Novel methodologies and strategies to promote nerve regeneration and functional recovery have been assayed, although their translation into clinical practice has been slow. These include the activation of intrinsic growth pathways, use of electrical and magnetic stimulation, optimization of nerve grafts, generation of biosynthetic grafts based on a variety of biomaterials, and grafting of stem cells and manufactured SCs for nerve repair [2].

Functional recovery is even more severely limited in the CNS, as in the case of spinal cord injury (SCI). This is due to the inhibitory milieu generated by oligodendrocytes, CNS glial cells, and their inability to support the growth of central axons. As a result, axonal elongation across the lesion fails. The CNS environment acts as a major barrier; reactive astrocytes secrete chondroitin sulfate proteoglycans (CSPGs) to form an impenetrable glial scar, and the oligodendrocytes release myelin-associated inhibitory molecules, such as Nogo-A and oligodendrocyte myelin glycoprotein (OMgp) [3]. Because nerve regeneration fails in the spinal cord, any observed functional recovery relies on adaptive synaptic plasticity. Uninjured circuits and local interneurons may reorganize, sprout new connections, and form detour circuits around the epicenter of the injury to bypass damaged pathways. These plasticity mechanisms and the potential exploitation of spinal networks supporting basic functions as locomotion and micturition provide the means for partial restoration after SCI.

Historically, the field of SCI repair has drawn inspiration from the known regenerative mechanisms of the PNS. Early pioneering work by Aguayo and colleagues demonstrated that transected central axons have an intrinsic capacity to elongate when provided with the permissive microenvironment of a peripheral nerve graft [4]. This evidence shifted the paradigm of spinal cord research from a doctrine of absolute regenerative failure to an active quest to replicate the conditions of the peripheral nerve within the injured spinal cord. Other pioneering repair strategies developed by the Bunge laboratory translated the concepts of tube repair and SC grafting, from bridging a gap in a PNI to repairing a SCI [5]. Indeed, the PNS and the spinal cord are functionally integrated during normal functioning as well as following focal injuries. A PNI always results in marked and durable modifications and reorganization in the spinal cord and the brain [6]. On the other hand, an SCI disconnects the neurons caudal to the injury, resulting in loss of function and target organ atrophy. The plasticity and reorganization of higher spinal and brain circuits are complex, including collateral sprouting, the unmasking of silent synapses and selective neuronal death. The reorganization of neural networks may result in beneficial adaptative changes or, contrarily, cause maladaptive changes that result in neuropathic pain, hyperreflexia and dystonia [7,8].

In this Special Issue of the *International Journal of Molecular Science*, entitled “Plasticity of the Nervous System after Injury: 2nd Edition” (https://www.mdpi.com/journal/ijms/special_issues/5Q4ECIITTU (accessed on 16 August 2026)), a series of eleven papers deal with a wide spectrum of topics related to either peripheral nerve or spinal cord injuries.

Traumatic peripheral nerve injuries frequently produce large defects in nerve continuity that preclude regenerating axons from reaching the denervated distal nerve and, thus, functional recovery. In the clinical setting, nerve autografts, allografts, and artificial devices are used to bridge the gap, even though they still have limitations. Nerve conduits composed of synthetic polymers or natural components of the extracellular matrix are a topic of intense research. Hollow conduits do support axonal growth but only up to a critical gap length, usually considered of 10 mm in the rat and 25–30 mm in human nerves. In this regard, advanced designs are made by combining an outer tube filled with aligned hydrogels or with microfibers.

The paper by Alves-Sampaio and Collazos [9] presents a detailed study in which they defined an optimal substrate and medium for growing dissociated SCs in culture. Then, PEDOT-coated carbon microfibers were covered with a complex of poly-L-lysine, heparin, basic fibroblast growth factor and fibronectin, resulting in enhanced SC proliferation and migration from nerve explants. Moreover, taking advantage of the electroconductive properties of the carbon microfibers, electrical stimulation was applied in the culture, producing a further increase in SC number and migration without favoring fibroblast proliferation. The results support the potential use of this dual function coated-carbon microfibers as a pro-regenerative scaffold for repairing in vivo nerve injuries.

One of the conduits that has reached the clinical market is made of chitosan, a natural biopolymer with beneficial properties for regenerative approaches. Preclinical studies reported an excellent outcome regarding nerve regeneration after repairing a critical nerve gap with a chitosan conduit [10]. A clinical trial to investigate the use of this conduit as a protective cover of end-to-end suture repair was pursued for digital nerves (Reaxon^®^ Direct, Medovent GmbH, Mainz, Germany) [11]. Focusing on the slow degradation rates of the initial chitosan conduits, Ronchi and colleagues [12] investigated the performance of two variants of chitosan conduits, one with slow degradation and one with faster degradation, that covered the end-to-end repair of the median nerve in adult rats. The results proved that the two conduit variants did not induce harmful tissue reaction or negatively impact the regenerated nerve. The slow degradation variant showed confirmed stability and flexibility over 6 months, and broke into longitudinal segments after 12 months. The fast variant showed considerable fragmentation as early as two months after implantation, with encapsulated chitosan pieces appearing near the implantation site. The authors recommend that chitosan conduits be applied only in locations where encapsulation of the degrading material has sufficient space to avoid irritating foreign-body-reactions in neighboring tissue.

The paper by Wood and colleagues [13] furthered their examinations of the limited growth of regenerating nerves across acellular nerve allografts, providing some evidence for the involvement of pro-inflammatory cytokines that increased with time. Their previous work had reported reduced regeneration through allografts, particularly long ones, associated with the appearance of ‘senescent’ SCs, identified by specific markers [14]. This current paper demonstrates that the proximal portion of the allograft was permissive of axonal regeneration but nerve regeneration in the mid-distal portion declined significantly over periods of up to 6 weeks. This decline occurred concurrently with a progressive increase in the expression of pro-inflammatory genes including IFN-γ and IL-6 in the mid-distal graft within long allografts. This was not caused by increased numbers of macrophages. Additionally, there were small changes in the morphology of the blood vessels. The authors did not consider a possible role of ischemia in reducing regeneration across long acellular nerve allografts. Perhaps their findings will be followed by studies to provide causal links between the declining regenerating axons through long allografts.

In contrast to the ability of rodent and pig SCs to myelinate regenerating axons in vivo and in vitro, human SCs fail to do so [15]. Miller/Kaplan laboratories initiated related studies when they isolated multipotent adult stem cells (iPSCs) from mammalian skin [16,17]. Chu and Midha [15] confirmed the ability of isolated SCs of rodent and pigs to myelinate neurites under several co-culture conditions. These SCs, however, showed limited myelination with both human iPSC-derived motoneurons and rodent dorsal root ganglion (DRG) neurons. The endogenous SCs in pre-degenerated human sciatic nerve segments from freshly harvested and autopsied donors, while supporting axonal regeneration through nerve grafts of NOD-SCID mouse sciatic nerve, did not contribute to their myelination. The authors considered, as a limitation in their study, that the iPSC-derived motoneurons may not fully replicate the maturity and complexity of primary human neurons. The authors surmised an intrinsic species-specific restriction rather than a cell culture-induced defect. They concluded that the translation of rodent-derived findings to humans is a remaining challenge. This conclusion is supported by their study and aligns with emerging evidence that highlights intrinsic interspecies differences in SC biology [18].

The immune cells, glial cells, and the microvasculature that comprise the microenvironment of peripheral nerves play key roles in the regenerative capacity of injured axons [19]. Malekzadeh et al. [20] reviewed this topic admirably. They first established the critical role of blood vessels that extend across a repair site in directing SC migration after the macrophage release of angiogenic factors (reviewed in [21]). Thereafter, current therapeutic approaches to peripheral nerve repair are the focus of the review after dispensing with the success of vascularized nerve grafts over long gaps (because there are too few appropriate donors), and considering acellular nerve allografts that have more advantages, such as their gradual cellularization by SCs and the extracellular matrix supporting axonal growth through the grafts. The considered experimental approaches include the following: (1) biomaterials such as fibronectin-laminin1-laminin 2 and fiber-reinforced 3D scaffolds with endothelial and SCs [22]; (2) the use of nanoparticles as carriers for bioactive molecules; (3) extracellular lipid-bound vesicles secreted by mesenchymal stem and umbilical endothelial cells, pericyte-derived nanovesicles, and SC-derived exosomes; (4) bioactive peptides such as VEGF, BDNF, and laminin; (5) viral and non-viral VEGF gene therapy to increase angiogenesis and nerve regeneration. Viral transfection, despite its high efficiency and stable gene expression, is limited by safety concerns, thereby increasing interest in non-viral methods of gene delivery.

The review of Verge et al. [23] provides a detailed description of the unfolded protein response (UPR) in the endoplasmic reticulum (ER), ER stress that results from elevated protein synthesis after PNI, and the essential role of Luman/CREB3 as a retrograde signal that transcriptionally regulates the beneficial adaptive UPR. This description precedes their focus on the potential of non-invasive therapeutic acute intermittent hypoxia (tAIH) treatment to improve recovery from nerve injury. The tAIH has been shown to have translational potential for SCI, organ protection, neurodegenerative disease and multiple sclerosis [24,25]. Verge et al. [23] compare the effects of AIH with the well-established effects of low-frequency electrical stimulation (ES) on peripheral nerve outgrowth and target reinnervation after nerve transection and repair [26]. The rationale for this comparison was that 1) AIH increased BDNF expression in motor and sensory neurons and 2) the upregulation of the transcription factor HIF1α by the low oxygen levels associated with nerve injury is increased further by ES and AIH [27]. They compare well in elevating early expression of regeneration-associated genes, increasing the number of neurons that regenerate their axons, and improving myelination of regenerating nerves. However, the links between Luman and its ability to regulate an adaptive UPR are weak currently.

Gordon [28] provided a detailed review of the conflicting views of the determinants of the neuromuscular properties of skeletal muscle and their recovery and plasticity after PNI. The conflicting view is that daily *amount* but *not* the *pattern* of neuromuscular activity determines muscle properties, as Vrbova and Pette had advocated strongly for 40 years [29,30]. In the 1990s, Eerbeek et al. [31] argued that the amount of daily neuromuscular activity was the determining factor on the basis of Henneman’s size principle of recruitment of motor units from the small–slow to the large–fast ones. Their experiments demonstrated that high daily activity converted fast skeletal muscles to slow whilst progressively lower levels of activity promoted fast-twitch muscle fibers [32,33]. Gordon and colleagues [34,35,36] then demonstrated that the amount of daily neuromuscular activity determines both the electrical properties of motoneurons and the contractile and metabolic properties of the muscle fibers that they supply.

This Special Issue includes two interesting papers that concern the regeneration and plasticity events that are implicated in SCIs, namely an investigation with a novel in vitro model and a study on the role of perineuronal nets in the plasticity processes.

The complex pathophysiology of SCI demands the establishment of adequate pre-clinical models to generate relevant mechanistic information and to serve as a screening platform for therapeutic agents. Most in vitro models have used cultures of only one cell type, mostly neurons [37]. Campos et al. [38] established a novel multicellular system that contained the major cell types from the spinal cord (neurons, astrocytes, microglia, and oligodendrocytes). These were obtained from extracted spinal cords of postnatal rats and cultured under conventional conditions. Then, a model of hypertonic osmotic injury was produced by adding sorbitol to the culture. This protocol induced marked phenotypic shifts in neuronal, astroglial, microglial, and oligodendrocytic populations. Specifically, about 50% of neurons died in the first 48 h of injury. This model can be used for the fast screening of neuroprotective drugs in order to design new therapies for application in SCI animal models that might improve functional outcomes. Indeed, as a proof-of-concept, the hyperosmotic model was used to study the neuroprotective impact of a human adipose tissue stem cell (hASC)-derived secretome, which has emerged as a potential therapeutic approach for SCI [39].

Perineuronal nets, mainly composed of hyaluronan, CSPGs, tenascin and link proteins, surround the soma of some populations of neurons, particularly spinal cord motoneurons. Perineuronal nets play an essential role in synaptic stabilization and in controlling plasticity in the CNS [40]. Interestingly, activity training differentially modulates perineuronal nets depending on whether an injury occurs in the spinal cord or in the brain. Sanchez-Ventura and coworkers [41] studied the activity-dependent modulation of spinal perineuronal nets, and found that voluntary running increased spinal perineuronal nets in wild-type (WT) mice but not in link protein 1 knock-out mice. This suggested that link protein 1 mediates the activity-dependent modulation of perineuronal nets. Following SCI, the decline in perineuronal nets due to the inactivity of spinal circuits was associated with the development of hyperreflexia and hyperalgesia at the end of the follow-up in WT-injured mice. In contrast, both hyperreflexia and hyperalgesia were already present before injury and remained unchanged after injury in link 1 KO mice. This was despite that both mice strains presenting with similar motor impairments. These findings demonstrate that link protein 1 participates in the correct formation as well as in the activity-dependent modulation of perineuronal nets, and offers an opportunity to modulate spinal cord plasticity after injury and prevent neuropathic pain and spasticity.

Finally, two review papers in this Special Issue present updated information that is relevant to both peripheral nerve and spinal cord injuries. The first one deals with the use of protein synthesis for restoring neural connectivity after axonal injury. The localization of mRNAs and translational machinery in axons and dendrites provide neurons with a renewable source of new proteins at distal sites. This in turn, enables a rapid modification of their proteomes that is independent of axonal transport from the soma. This is particularly important in initiating and sustaining axon regeneration following injury [42], in which the arrival of positive injury signals, synthetized at the lesion site at the neuronal soma, induces the expression of genes involved in cell survival and axonal growth. Twiss and Buchanan [43] summarize critical post-transcriptional regulatory mechanisms, emphasizing translation in the axonal compartment, and highlighting potential strategies for designing regeneration-promoting treatments. Some interesting points come from studies evaluating axonal transcriptomes as well as from the approaches used to isolate translationally active mRNAs from axons (the ‘axonal translatome’). One example is the functions of locally synthesized axonal proteins, which include contributions to axon growth, the axonal injury response, nuclear transcriptional regulation, retrograde transport, and vesicle and mitochondrial transport. In mature PNS axons, axotomy triggers the rapid and localized translation of stored mRNAs. Axonal proteins synthesized in the early stages after injury are used for retrograde signaling and for initiating growth cone formation. They also review the environmental stimuli in the growth-permissive environment of the peripheral nerve that further increase the translation of growth-associated mRNAs. The requirement for axonal synthesized proteins in the early stages of regeneration highlights several promising therapeutic targets to accelerate axonal regeneration.

The second review, by Palomés-Borrajo et al. [44], is dedicated to the role of epigenetic modifications, particularly histone acetylation, in modulating gene expression programs that underly axonal regeneration after both PNI and SCI, and how understanding these epigenetic mechanisms may lead to new strategies for enhancing axonal growth and plasticity. The roles of epigenetic writers (histone acetyltransferases, HAT), erasers (histone deacetylases, HDAC) and readers are explored with regard to their effects on different types of neurons. Indeed, HATs like p300/CBP increase the expression of several regeneration-associated genes in DRG neurons as well as in spinal cord sensory neurons, but not in corticospinal neurons. On the other hand, treatment with HDAC-inhibitor compounds has been shown to facilitate neurite outgrowth, neuroprotection, and functional recovery in both PNS and CNS injury models. Among the epigenetic readers, special attention is paid to the BET proteins family. BET protein inhibition improved neuroprotection and decreased inflammation and, in turn, the functional outcome in mice subjected to spinal cord contusion [45]. However, the same compound was ineffective in improving outcomes after nerve injury and reduced neurite length in DRG explants in vitro. Interestingly, this paper also includes a section on the impact of epigenetic modifiers after nerve injuries on non-neuronal cells, including microglia/macrophages, SCs and oligodendrocytes, and astroglia. These are relevant when interpreting the findings from in vitro experiments on isolated neurons for translation to in vivo trials.

## 2. Summary

The aim of this Special Issue is to increase interest among a wide interdisciplinary audience of neuroscientists, from molecular biologists to clinical researchers, to promote scientific progress in the field of peripheral nerve and spinal cord injuries. The articles included in this Issue cover wide-ranging perspectives on injuries to the nervous system and present new approaches to promote recovery.
